# Three-dimensional structured illumination microscopy data of mitochondria and lysosomes in cardiomyoblasts under normal and galactose-adapted conditions

**DOI:** 10.1038/s41597-022-01207-7

**Published:** 2022-03-23

**Authors:** Ida S. Opstad, Gustav Godtliebsen, Florian Ströhl, Truls Myrmel, Balpreet Singh Ahluwalia, Krishna Agarwal, Åsa Birna Birgisdottir

**Affiliations:** 1 Department of Physics and Technology, UiT The Arctic University of Norway, Tromsø, 9019 Norway; 2 Department of Clinical Medicine, UiT The Arctic University of Norway, Tromsø, 9019 Norway; 3 Department of Clinical Science, Intervention and Technology, Karolinska Institute, Stockholm, Sweden; 4 University Hospital of North Norway, Tromsø, 9019 Norway

**Keywords:** Super-resolution microscopy, Mitochondria

## Abstract

This three-dimensional structured illumination microscopy (3DSIM) dataset was generated to highlight the suitability of 3DSIM to investigate mitochondria-derived vesicles (MDVs) in H9c2 cardiomyoblasts in living or fixed cells. MDVs act as a mitochondria quality control mechanism. The cells were stably expressing the tandem-tag eGFP-mCherry-OMP25-TM (outer mitochondrial membrane) which can be used as a sensor for acidity. A part of the dataset is showing correlative imaging of lysosomes labeled using LysoTracker in fixed and living cells. The cells were cultivated in either normal or glucose-deprived medium containing galactose. The resulting 3DSIM data were of high quality and can be used to undertake a variety of studies. Interestingly, many dynamic tubules derived from mitochondria are visible in the 3DSIM videos under both glucose and galactose-adapted growth conditions. As the raw 3DSIM data, optical parameters, and reconstructed 3DSIM images are provided, the data is especially suitable for use in the development of SIM reconstruction algorithms, bioimage analysis methods, and for biological studies of mitochondria.

## Abstract (summary)

**Table:** 

Measurement(s)	fluorescence microscopy images of mitochondria
Technology Type(s)	three dimensional structured illumination microscopy
Sample Characteristic - Organism	Rattus norvegicus • H9c2 cardiomyoblast cell-line

## Background & Summary

Fluorescence microscopy is a popular and powerful method of achieving contrast and specificity in bioimaging experiments^1^. The obtainable resolution, however, has a theoretical maximum set by the Abbe resolution limit^2^, although other important factors are the quality of the sample and imaging system which usually further limit the achieved resolution^3^.

Optical microscopy techniques that can achieve a resolution beyond the conventional limit are called super-resolution microscopy (SRM)^4^. The enhanced resolution can be achieved through both optical system design and/or computational post-processing. Further, the acquired data must also generally follow high standards and there are specific requirements of the samples subjected to imaging with respect to the fluorescent markers and imaging conditions. A deviation from the required high data standards with respect to critical factors like optical system alignment, suitability of the fluorescent marker, and overall imaging conditions, can lead to an image quality inferior to that achievable using conventional fluorescence microscopy^5^.

Three-dimensional structured illumination microscopy (3DSIM) is an SRM technique requiring both the use of engineered illumination patterns and a reconstruction algorithm. It is a wide-field imaging technique achieving resolution doubling in three dimensions compared to the aforementioned Abbe resolution limit^6^. Relative to other SRM techniques, it is fast and gentle with respect to illumination intensities and requirements of fluorescent markers and imaging buffers^4^. Further, it has proven suitable for multi-color super-resolution imaging of mitochondria in three-dimensions^7^.

The main challenges in SRM beyond the strict sample and imaging system requirements involve phototoxicity, photobleaching, imaging speed, throughput, reconstruction artifacts, data analysis, and the validation of results^4,8^. A major challenge for the developers of reconstruction algorithms and computational methods is the lack of suitable data, as instruments for SRM or the required interdisciplinary knowledge and training necessary for generating good bioimage datasets are to most researchers not readily available. Usually, the complete data following publication related to SRM is not provided^9^. The provision of only compressed illustrations or the lack of metadata are related and important issues hindering the reuse of research data^10^.

**Fig. 1:**
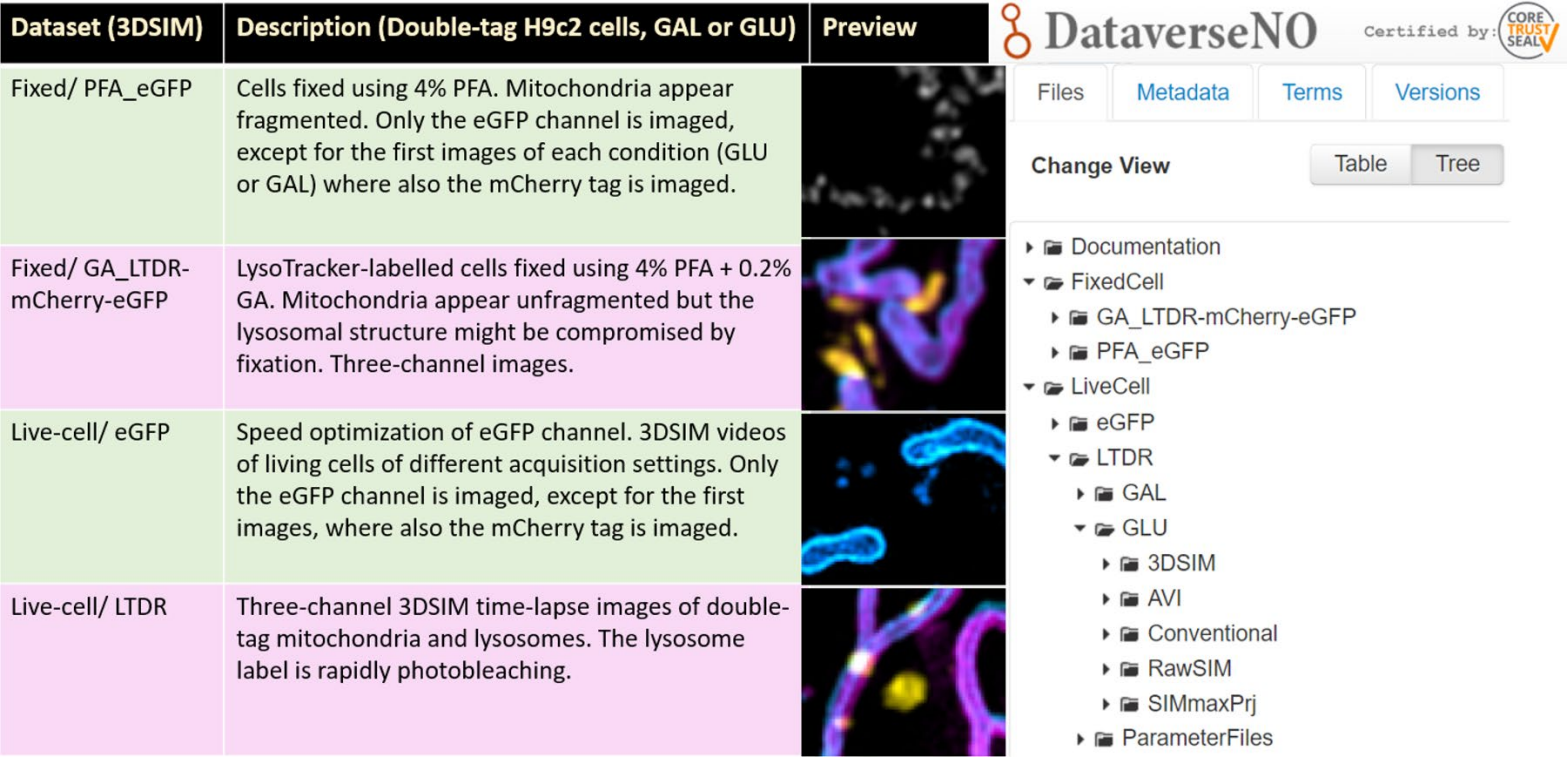
Overview of 3DSIM datasets. All data is of the cardiomyoblast cell-line H9c2 stably labeled with the tandem-tag eGFP-mCherry-OMP25-TM (an acidity indicator). The cells are cultivated under either normal conditions with glucose (GLU) or without glucose, adapted to galactose (GAL). The images are acquired under four different sample conditions: PFA-fixed, PFA + GA-fixed (labeled with LTDR), living cells, and living cells with LTDR. The preview column displays snapshots of the different samples (z-projected 3DSIM images, pseudo-colored using Fiji). The right column shows how the dataset appears on DataverseNO in Tree View.

The current dataset – outlined in Fig. 1 – was initially acquired to investigate the suitability of 3DSIM for two different biological applications: a) the study of mitochondria-derived vesicles (MDVs) in living or fixed samples, and b) correlative imaging of the tandem-tag acidity sensor mCherry-eGFP with LysoTracker Deep Red (LTDR), both in the cardiomyoblast cell-line H9c2 with a stable expression of mCherry-eGFP fluorescent-tagged trans-membrane domain of the outer mitochondria membrane protein 25 (OMP25). As the mCherry fluorescent protein is acid-stable (pKa < 4.5) while the eGFP is acid-labile (pKa = 6.0) and rapidly quenched in an acidic environment, this tandem-tag construct can be used as an acidity sensor and assay for lysosomal degradation of mitochondria^11^. MDVs act in mitochondria quality control and are small (70–150 nm in diameter), single or double-membrane vesicles that arise through budding from the mitochondria^12^.

Images of cells from two different growth conditions are provided: in normal media containing glucose, or in glucose-free, galactose-adapted growth conditions. Further, two different imaging conditions are provided for both live- and fixed-cell imaging: living cells with or without LTDR staining, and fixed cells chemically preserved using either paraformaldehyde (PFA) or PFA in combination with glutaraldehyde (GA). This extensive and high-quality 3DSIM dataset is provided as a community resource intended for those working on 3DSIM reconstructions, bioimage analysis, or mitochondria research.

## Methods

### Cell-culture and sample preparation

The rat cardiomyoblast cell-line H9c2 (cells derived from embryonic heart tissue; Sigma Aldrich) was genetically modified using retrovirus to achieve a stable expression of tandem tagged (mCherry-eGFP) mitochondrial outer membrane protein 25 (OMP25)-transmembrane domain (TM). A uniform expression of fluorescence intensity in the cells was achieved through flow cytometry sorting. The stable H9c2 cells were cultured in high glucose (4.5 g/L) Dulbecco’s Modified Eagle Medium (DMEM, D5796, Sigma-Aldrich) with 10% FBS, 1% streptomycin/penicillin and 1 µg/ml of puromycin (InvivoGen). For glucose deprivation and adaptation to galactose, the cells were grown in DMEM without glucose (11966-025, Gibco) supplemented with 2 mM L-glutamine, 1 mM sodium pyruvate, 10 mM galactose, 10% FBS, 1% streptomycin/penicillin, and 1 µg/ml of puromycin (InvivoGen). The cells were adapted to galactose for a minimum of 7 days before experiments. The cells were seeded on MatTek dishes (P35G-1.5-14-C, MatTek Corporation) and imaged when they reached approximately 80% confluency.

**Labeling of lysosomes** (acidic endosomal system) was done by incubating the cells for 40 minutes with 100 nM LysoTracker Deep Red (LTDR, Cat nr L12492, Thermo Fisher) in the normal cell-culture medium (with either glucose or galactose). After labeling, the media was replaced with fresh cell-culture media right before live imaging.

#### Cell fixation

The fixed samples were treated as above, but fixed using either 4% paraformaldehyde (PFA) or 4% PFA + 0.2% glutaraldehyde (GA) in phosphate-buffered saline (PBS, preheated to 37°C) for about $30$ min at room temperature. The samples were then washed and re-immersed in PBS before imaging. Only the GA samples were labeled using LTDR.

#### Imaging conditions

The living cells were imaged in their usual growth medium at 37°C with atmospheric gas levels. The fixed samples were imaged at either room temperature or at 37°C in PBS.

### Microscope

The images were acquired using a DeltaVision OMX V4 Blaze imaging system (GE Healthcare) equipped with a 60X 1.42NA oil-immersion objective (Olympus), three sCMOS cameras, and 405, 488, 568, and 642 nm lasers for excitation. The vendor-specified optical resolution of the 3DSIM system is 110–160 nm laterally, and 340–380 nm axially, depending on color channel. To surpass the diffraction limit, this SIM set-up uses sinusoidal illumination patterns and acquires 120 images per 1 µm z-stack thickness (3 illumination angles times 5 phase shifts times 8 planes/µm thickness) per color channel. Super-resolution 3D images are then obtained via image processing using the reconstruction software described under Image processing.

### Image processing

#### Image reconstruction and channel registration

Image deconvolution and 3DSIM reconstructions were completed using the manufacturer-supplied softWoRx program (GE Healthcare). Image registration (color channel alignment) was also performed in the same program using experimentally-measured calibration values compensating for minor lateral and axial shifts, rotation, and magnification differences between cameras. The pixel area of the 3DSIM images is 1/4 of the acquired raw data (40 nm by 40 nm vs. 80 nm by 80 nm). The files in the SIMmaxPRj folders were additionally maximum intensity z-projected as a final step.

#### Image analysis and processing

Image analysis and processing beyond the preprocessing described above was done using Fiji^13^. This includes the conversion from DV to TIFF image files (preserving bit-depth and metadata), the generation of AVI movies (maximum intensity z-projected and bleach corrected image sequenced using the exponential fit option), and visualization using orthogonal views.

## Data Records

The data is published on the DataverseNO repository in the UiT Open Research Data collection 10.18710/PDCLAS^14^. The image files were originally collected as DV files but were losslessly (preserving bit depth and metadata) converted to TIFF (either 16- or 32-bit hyperstacks) before archiving. For each collected image file or image reconstruction, there is an associated text file describing the acquisition parameters. Records of the image acquisition are also available in the image metadata (in ImageJ/Fiji, the metadata can be read using the keyboard shortcut “I”).

In addition to a description of the overall dataset and folders, an explanation of how the **file naming convention** translates back to experimental conditions is available in the ReadMe file associated with the published dataset.

To achieve a successful upload to the data repository, the data was stored (losslessly) in ZIP archives. A complete list of the files contained within the different ZIP files is provided in Documentation/00_FileOrganization.txt.

### Data folders and experimental conditions

The data is collected into four main imaging experiments as summarized in Table 1. Each of the main folders contains images of cells from both the GAL and GLU growth conditions (indicated in the file names):

- The **Fixed/PFA_eGFP** data is of cells fixed using 4% PFA in PBS. The data was found non-suitable for MDV quantification due to the fragmentation of mitochondria. Most of the images are of the eGFP channel only.
- The **Fixed/GA_LTDR-mCherry-eGFP** data are samples prepared the same way as for Live-cell/_LTDR, but fixed using 4% PFA + 0.2% GA in PBS. The mitochondria no longer appear fragmented as for Fixed/PFAe_eGFP, but with a higher autofluorescence due to the GA treatment, which can cause some additional SIM reconstruction artifacts.
- The **Live-cell/eGFP** data contains 3DSIM time-lapses of mitochondria (eGFP channel only, except for the first images) of gradually faster acquisition rate and more time-points per file towards the higher appended image numbers. Several different acquisition parameters are used, especially concerning image size and camera read mode.
- The **Live-cell_LTDR** data are three-channel 10 s time-lapses (10 time-points each) of both mitochondrial tags and the lysosomal marker LTDR.

**Table 1:** The entire dataset is of the cardiomyoblast cell-line H9c2 (derived from rat), stably expressing the tandem-tag mCherry-eGFP on mitochondria (OMP25-TM).

Data	Live/fixed	Labels	Notes
Fixed/PFA_eGFP	PFA	mCherry-eGFP	Fragmented mitochondria
Fixed/GA_LTDR-mCherry-eGFP	PFA + GA	mCherry-eGFP + LTDR	Three-channel images
LiveCell/eGFP	Live	mCherry-eGFP	488 nm excitation; fast time-lapse imaging
LiveCell/LTDR	Live	mCherry-eGFP + LTDR	10 s time-lapses; photobleaching

Within the four folders of different imaging conditions, data from additional two different growth conditions (GAL and GLU) are provided.

## Technical Validation

### Imaging system and data acquisition

**Fig. 2:**
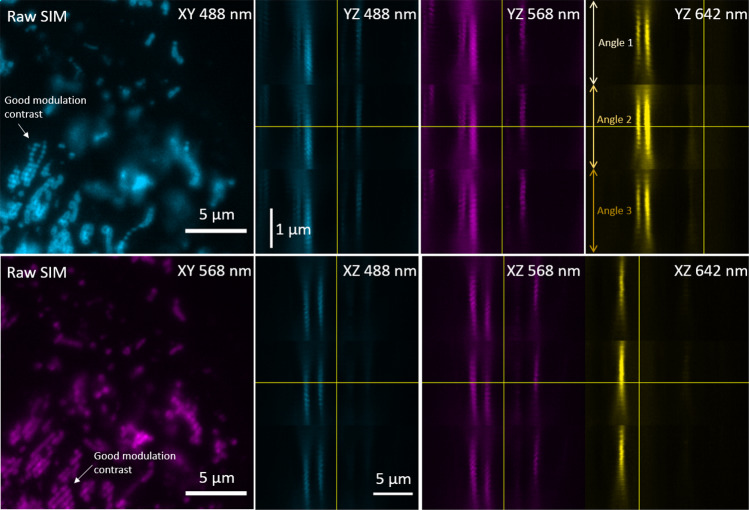
High-quality structured illumination data is characterized by good modulation contrast, display similar intensity strength for all illumination angles, only minor levels of photobleaching (<10% over one 3D image stack), and has a symmetrical signal spread in the axial direction (the immersion oil refractive index – 1.520 in this case – is adjusted for small sample variations to obtain a symmetrical PSF). The left panels show examples of good SIM data (single plane, excitation 488 nm and 568 nm) with a clear stripe pattern in the in-focus areas. The panels on the left show data from the same experiment, but the entire dataset (single time-point) in orthogonal view. The yellow lines indicate the image center for each dimension. The images are sorted after illumination angle, such that the 2 µm z-stack is scanned through trice. For each illumination angle, there are five phase shifts repeated for 16 z-planes (8 per µm).

The commercial OMX Blaze 3DSIM imaging system was installed and quality checked by a system engineer. The image acquisition, optimization of immersion oil and reconstruction quality was overseen by an expert user. Further, and according to individual needs, both raw and reconstructed 3DSIM data can be checked for particular features and quality measures using the open-source Fiji plugin SIMcheck^15^. The main characteristics of good SIM raw data are outlined in Fig. 2.

### Image quality

**Fig. 3:**
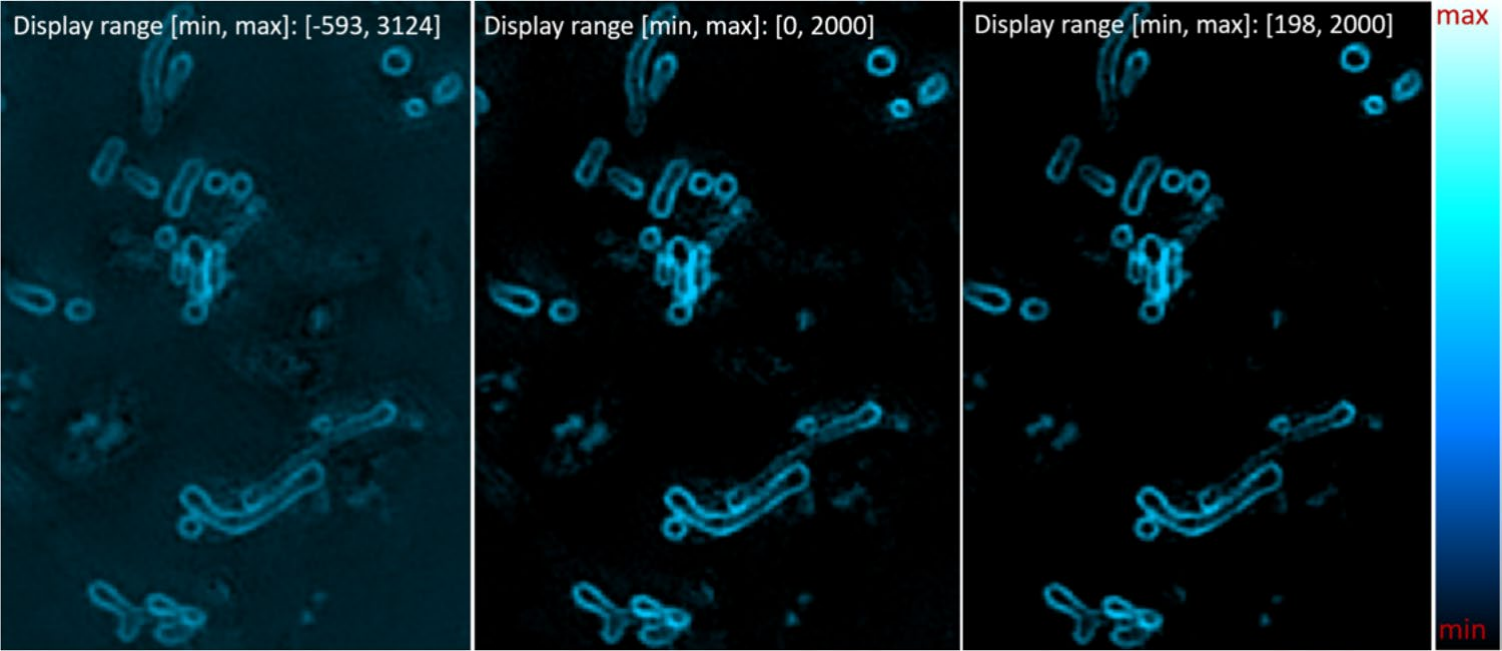
A good SIM reconstruction has reconstruction artifacts only of intensities significantly below that of the biological features of interest. The panels show a single plane SIM image with different linear intensity scaling: all values shown from −593 (negative intensities sometimes result from SIM reconstruction), lowest displayed intensity 0, and with the lowest displayed intensity 198. Most reconstruction artifacts and background haze is already removed from the image by discarding the negative intensity values. A few remaining image features not well reconstructed can here be further filtered out by disregarding intensities up to 198, as the well-reconstructed biological features are of significantly higher intensities.

Although of overall good quality, SIM images can never be assumed to be completely without reconstruction artifacts. Due to cell and sample variability, some variation in image quality is also expected, even for the same sample and identical acquisition parameters. When the SIM artifacts are of significantly lower intensity values than the biological features of interest, such that the artifacts can be removed by linearly adjusting the image brightness, we consider the image quality good. The nonphysical negative intensity values resulting from the reconstruction algorithm can usually be discarded without losing biological context. This is illustrated in Fig. 3. Recognizing what are SIM artifacts or actual sample features can require some training. Common artifacts, their causes together with different trade-offs in SIM acquisition are explained by Demmerle *et al*.^16^.

### Cell-line

For retroviral-based creation of stable cells with constitutive expression of fluorescent mitochondria marker, we used the commercially available and authenticated cell-line H9c2 (2-1) Rat DB1X heart myoblast (Catalogue No: 88092904; Lot No. 08G008 P + 8; European Collection of Authenticated Cell Cultures; Distributed by Sigma Aldrich, Norway). To ensure uniform fluorescent marker expression, the cells were sorted using flow cytometry. The cells tested negative for mycoplasma.

### Sample quality after aldehyde fixation

The main goal of sample fixation in microscopy is to provide a snapshot of the sample morphology without the effects of motion blur or phototoxicity during imaging, cellular changes due to environmental changes or just the duration of time between sample preparation and imaging. What is a suitable fixation protocol depends on the sample as well as the imaging method and desired analysis^17,18^. For our cells with mitochondria-derived (nano-) vesicles as the target of our study, we first opted for fixation using 4% PFA in PBS. At 3DSIM resolution and with the desire of quantifying nanovesicles, this fixation method was deemed unsuitable for organelle preservation because of clear fragmentation of mitochondria obstructing reliable quantification of small vesicles. Using instead a combination of PFA and GA preserved the live-cell morphology much better. This is nicely illustrated in Fig. 2 of reference^19^ using the same dataset. It also shows a comparison of a conventional (deconvolved) image, where the difference in live vs. fixed cell morphology is much harder to detect and thus might be less significant for other types of imaging techniques and analysis. A downside of the GA-based fixation is that GA itself exhibits a strong fluorescence^20^. This causes unspecific fluorescence which can affect the SIM reconstruction quality. We found 0.2% GA together with 4% PFA to be a suitable compromise for our application.

### Biological context

Glucose serves as the primary metabolic fuel of mammals^21^. Glucose is converted to energy in the form of adenosine triphosphate (ATP) in a process termed glycolysis. The main ATP energy production in normal cells however, is conducted in the mitochondria through oxidative phosphorylation (OXPHOS). H9c2 cells under normal culture conditions with high glucose levels primarily utilize glycolysis for ATP generation. In contrast, H9c2 cells deprived of glucose and adapted to galactose rely entirely on OXPHOS to meet their energy requirements. As a consequence, glucose deprivation has been shown to render the cells more sensitive to mitochondrial damage^22^. This is likely to cause differences between cells under the two different cultivation conditions, although we have noted also a large variation within the two groups when it comes to e.g., mitochondrial morphology, amount of lysosomes, and amount of mitochondrial fragments found in lysosomes. In reference^19^, the glucose-deprived cells were shown to contain more mitochondria-derived vesicles than the cells given glucose (i.e., normal growth conditions).

## Usage Notes

The images (TIFF and OTF files) can be opened in ImageJ/Fiji or other programs for image processing. Please be aware of the negative intensities in the 3DSIM reconstruction. The negative intensities are a peculiarity often resulting from the SIM reconstruction process. Although an undesirable image artifact, keeping the negative intensities can be important for checking the reconstruction quality of SIM data^15^ and for recognizing artifacts by visual inspection^16^. For most purposes, the negative intensities can be set to zero for visualization and before analysis (e.g. in Fiji, using the Brightness and Contrast tool). The log or image registration files (txt or dat) can be opened using any text editor.

An overview of the image content, quality and biological events of the larger 3DSIM time-lapse files of **LiveCell** can be quickly accessed through the AVI_movies folder. Here, events like extending mitochondria-derived tubules or MDV dynamics can be observed. Similarly, the files in SIMmaxPrj can also be used as a single-plane visualization tool to get an overview of the data without downloading or opening the entire 3DSIM files. The Conventional folders contain conventional fluorescence microscopy data, deconvolved images, and transmission microscopy images as a reference for the super-resolution microscopy data. Images depicting special cases of mitochondria away from their normal confinement inside cells are provided by 20210511_H9c2-dTag_GAL_PFA4-GA02_LTDR100nm-40m_1520_con_001 (conventional image in Fixed/GA_LTDR-mCherry-eGFP/GAL) and 20210511_H9c2-dTag_GAL_LTDR100nm-40m_1520_sim256_013 in LiveCell/LTDR/GAL (challenging 3DSIM data). These files should be discarded from analysis concerning, e.g., the number or morphology of mitochondria and lysosomes inside cells.

The experimental meaning of folder and file names are explained in the dataset’s ReadMe file (in the DATA & FILE OVERVIEW section). The experimental settings for each image file can be accessed without opening the images via the text files in the RawSIM_log folders. Details of the 3DSIM reconstructions conducted are available in the reconstruction log files in the 3DSIM/SIRlog folder. The parameter files used for the 3DSIM reconstructions and for the channel registration/alignment by the commercial softWoRx program are provided in the Parameterfiles folders. These contain experimental point spread functions (PSFs, OTF files) for three different wavelengths (green, red, and far red channels) and image registration data (dat file). The point spread functions describe the imaging systems’ response to a point emitter, or for this experimental case, to a 100 nm fluorescent bead (of different emission wavelengths).

The LiveCell/LTDR and Fixed/GA_LTDR-mCherry-eGFP data are best suited for comparing the GLU and GAL growth conditions. Note, however, the rapid photobleaching of LTDR.

Apart from biological inferences, the long time-lapses of the Live-cell data might be interesting 3DSIM data for testing the performance of new reconstruction algorithms, as the signal-to-noise ratio (and the 3DSIM image quality) goes down towards the end.

### Acquisition speed

The additional filename annotations *fast, faster, fasterer* for the LiveCell/eGFP experiments indicate changes in the acquisition settings achieving gradually faster acquisition rate, i.e., changes in camera mode, camera read area, exposure time, stack size and time-lapse (i.e., additional time-delay added between each time-point other than single-image acquisition time). Only one setting was changed at the time such that the effect could be readily assessed by comparing different experiments (i.e., image files). The achieved acquisition rate for the LiveCell/eGFP experiments is in the range 1 s – 5 s per 3DSIM volume. For the particular example displayed in Fig. 1^19^, the 3DSIM frame rate was 1.5 s (file: 20210420_H9C2-dTag_GAL_37C_1520_sim-fasterer_018_SIR_PRJ).

The acquisition settings are most reliably read from the associated image log files (e.g. in LiveCell/eGFP/RawSIM/log), but can in some cases be found in the image metadata as well. The actual resulting acquisition time for an image frame (which can be significantly different from the targeted *Time-lapse* setting) can be read from the image metadata as timestamps. For multi-channel data, W0, W1, W2, refer to the acquisition of three different color channels (e.g., Cy5, A568, GFP), which have different timestamps as sequential acquisition mode was used. The example *Extended header Z0 W2 T0:timeStampSeconds* = *5.143928*, means that this was the first z-plane in the image stack, the third color channel, and the first time-point acquired after 5.15 seconds.

Tests of acquisition speed and parameters were also conducted for the LiveCell/LTDR multi-channel experiments. The first images of GLU (name containing 002, 003, 004, 005) are acquired using different parameters than the rest. 002 and 003 were acquired using higher illumination intensities for the remaining data (30% transmission (%T) rather than 10%T for the GFP and A568 channels, see the acquisition log files). 004 is only one frame, 005 differs from the rest (006–016 in GLU and 003–012 in GAL) only by *Time-lapse* being set to 3 s instead of 5 s, but resulting in about the same recorded time (70.5 s for 10 three-channel 3DSIM volumes) as the microscope cannot image any faster with the experimental settings given. For the file 017 in the same folder, the time-lapse settings were 10 s. Here, the time between each recorded time-point is 10 s.

## Data Availability

The 3DSIM reconstructions, image deconvolutions and associated image registrations were completed using the proprietary software softWoRx 7.0.0 following the microscope.

## References

[1] Sanderson MJ, Smith I, Parker I, Bootman MD (2014). Fluorescence microscopy. Cold Spring Harbor Protocols.

[2] Abbe E (1873). Contributions to the theory of the microscope and that microscopic perception. Arch. Microsc. Anat.

[3] Jost AP-T, Waters JC (2019). Designing a rigorous microscopy experiment: Validating methods and avoiding bias. Journal of Cell Biology.

[4] Schermelleh L (2019). Super-resolution microscopy demystified. Nature cell biology.

[5] Lambert TJ, Waters JC (2017). Navigating challenges in the application of superresolution microscopy. Journal of Cell Biology.

[6] Shao L, Kner P, Rego EH, Gustafsson MG (2011). Super-resolution 3d microscopy of live whole cells using structured illumination. Nature methods.

[7] Opstad IS, Wolfson DL, Øie CI, Ahluwalia BS (2018). Multi-color imaging of sub-mitochondrial structures in living cells using structured illumination microscopy. Nanophotonics.

[8] Tosheva KL, Yuan Y, Pereira PM, Culley S, Henriques R (2020). Between life and death: strategies to reduce phototoxicity in super-resolution microscopy. Journal of Physics D: Applied Physics.

[9] Pospíšil J (2019). Imaging tissues and cells beyond the diffraction limit with structured illumination microscopy and bayesian image reconstruction. Gigascience.

[10] Williams E (2017). Image data resource: a bioimage data integration and publication platform. Nature methods.

[11] Pankiv S (2007). p62/sqstm1 binds directly to atg8/lc3 to facilitate degradation of ubiquitinated protein aggregates by autophagy. Journal of biological chemistry.

[12] Sugiura A, McLelland G-L, Fon EA, McBride HM (2014). A new pathway for mitochondrial quality control: mitochondrial-derived vesicles. The EMBO journal.

[13] Schindelin J (2012). Fiji: an open-source platform for biological-image analysis. Nature methods.

[14] Opstad IS (2021). DataverseNO.

[15] Ball G (2015). Simcheck: a toolbox for successful super-resolution structured illumination microscopy. Scientific reports.

[16] Demmerle J (2017). Strategic and practical guidelines for successful structured illumination microscopy. Nature protocols.

[17] Hoetelmans RW (2001). Effects of acetone, methanol, or paraformaldehyde on cellular structure, visualized by reflection contrast microscopy and transmission and scanning electron microscopy. Applied Immunohistochemistry & Molecular Morphology.

[18] Villegas-Hernández LE (2020). Visualizing ultrastructural details of placental tissue with super-resolution structured illumination microscopy. Placenta.

[19] Opstad, I. S. *et al*. Mitochondrial dynamics and quantification of mitochondria-derived vesicles in cardiomyoblasts using structured illumination microscopy. *Journal of Biophotonics* e202100305 (2021). 10.1002/jbio.202100305 34766731

[20] Abay A (2019). Glutaraldehyde–a subtle tool in the investigation of healthy and pathologic red blood cells. Frontiers in physiology.

[21] Nakrani, M. N., Wineland, R. H. & Anjum, F. Physiology, glucose metabolism. *StatPearls [Internet]* (2021). 32809434

[22] Dott W, Mistry P, Wright J, Cain K, Herbert KE (2014). Modulation of mitochondrial bioenergetics in a skeletal muscle cell line model of mitochondrial toxicity. Redox biology.

